# Is it new or wrong? Responsibility in reporting novel structures.

**DOI:** 10.1063/4.0000828

**Published:** 2025-10-27

**Authors:** Glenn P Yap

**Affiliations:** University of Delaware, Newark, DE, USA

## Abstract

X-ray structures that reinforce precedent can assure the researcher that perhaps there is some understanding and predictability. Unusual structures often prove exciting since they could uncover new knowledge. An unusually short, at the time of reporting, Cr-Cr bond and a "starfish" crown ether structure which was reinterpreted with additional data unavailable at the time of the original report, will be briefly reviewed. Reporting a structure that bucks the trend with less-than-ideal data presents the crystallographer with a dilemma. Does one withhold discovery amidst concerns of the potential misleading conclusions and academic embarrassment or attempt publication albeit with caveats and disclosures in hopes of promoting discussion? An X-ray structure of a coordination compound that resists depositing as a good quality crystal shows a coordination mode that the first example of a metal tetrapyrrole complex featuring two supporting dicarbonyl ligands in a cis arrangement.

